# Shoulder dystocia in babies born to Aboriginal mothers with diabetes: a population-based cohort study, 1998–2015

**DOI:** 10.1186/s12884-024-06484-1

**Published:** 2024-05-30

**Authors:** Marwan Awad Ahmed, Helen D. Bailey, Gavin Pereira, Scott W. White, Kingsley Wong, Rhonda Marriott, Matthew J. L. Hare, Bridgette J. McNamara, Carrington C. J. Shepherd

**Affiliations:** 1 Telethon Kids Institute, University of Western Australia, Perth, Australia; 2 School of Population and Global Health, The University of Western Australia, Perth, Australia; 3 Curtin Medical School, Faculty of Health Sciences, Curtin University, Perth, Australia; 4 Curtin School of Population Health, Curtin University, Perth, Western Australia Australia; 5 Centre for Fertility and Health (CeFH), Norwegian Institute of Public Health, Oslo, Norway; 6 enAble Institute, Curtin University, Perth, Western Australia Australia; 7 Division of Obstetrics and Gynaecology, The University of Western Australia, Perth, WA Australia; 8 Maternal Fetal Medicine Service, King Edward Memorial Hospital, Subiaco, WA Australia; 9 Ngangk Yira Research Centre, Murdoch University, Perth, WA Australia; 10 Wellbeing and Chronic Preventable Diseases Division, Menzies School of Health Research, Charles Darwin University, Darwin, Northern Territory Australia; 11 Department of Endocrinology, Royal Darwin Hospital, Darwin, Northern Territory Australia; 12 Melbourne School of Population and Global Health, The University of Melbourne, Parkville, Australia; 13 Barwon South West Public Health Unit, Barwon Health, Geelong, Victoria Australia

**Keywords:** Aboriginal, Shoulder dystocia, Diabetes in pregnancy

## Abstract

**Background:**

Australian Aboriginal and Torres Strait Islander women with diabetes in pregnancy (DIP) are more likely to have glycaemic levels above the target range, and their babies are thus at higher risk of excessive fetal growth. Shoulder dystocia, defined by failure of spontaneous birth of fetal shoulder after birth of the head requiring obstetric maneuvers, is an obstetric emergency that is strongly associated with DIP and fetal size. The aim of this study was to investigate the epidemiology of shoulder dystocia in Aboriginal babies born to mothers with DIP.

**Methods:**

Stratifying by Aboriginal status, characteristics of births complicated by shoulder dystocia in women with and without DIP were compared and incidence and time-trends of shoulder dystocia were described. Compliance with guidelines aiming at preventing shoulder dystocia in women with DIP were compared. Post-logistic regression estimation was used to calculate the population attributable fractions (PAFs) for shoulder dystocia associated with DIP and to estimate probabilities of shoulder dystocia in babies born to mothers with DIP at birthweights > 3 kg.

**Results:**

Rates of shoulder dystocia from vaginal births in Aboriginal babies born to mothers with DIP were double that of their non-Aboriginal counterparts (6.3% vs 3.2%, *p < *0.001), with no improvement over time. Aboriginal mothers with diabetes whose pregnancies were complicated by shoulder dystocia were more likely to have a history of shoulder dystocia (13.1% vs 6.3%, *p* = 0.032). Rates of guideline-recommended elective caesarean section in pregnancies with diabetes and birthweight > 4.5 kg were lower in the Aboriginal women (28.6% vs 43.1%, *p* = 0.004). PAFs indicated that 13.4% (95% CI: 9.7%-16.9%) of shoulder dystocia cases in Aboriginal (2.7% (95% CI: 2.1%-3.4%) in non-Aboriginal) women were attributable to DIP. Probability of shoulder dystocia among babies born to Aboriginal mothers with DIP was higher at birthweights > 3 kg.

**Conclusions:**

Aboriginal mothers with DIP had a higher risk of shoulder dystocia and a stronger association between birthweight and shoulder dystocia. Many cases were recurrent. These factors should be considered in clinical practice and when counselling women.

**Supplementary Information:**

The online version contains supplementary material available at 10.1186/s12884-024-06484-1.

## Introduction

Australian Aboriginal and Torres Strait Islander people, like Indigenous populations globally [1], have a heavier burden of diabetes in pregnancy (DIP) [2, 3]. Aboriginal pregnant women may experience 10 times higher rates of type 2 diabetes and 1.5 times higher rates of gestational diabetes mellitus (GDM) compared to their non-Aboriginal counterparts [4]. Moreover, the burden of DIP among the Aboriginal population is substantially growing over time [3].

DIP is associated with significant adverse maternal and neonatal outcomes [5], contributing to the disparities between Aboriginal and non-Aboriginal populations. DIP is associated with perinatal death, preterm birth, excessive or restricted fetal growth, congenital anomalies, respiratory distress syndrome and shoulder dystocia [5].

Shoulder dystocia is an obstetric emergency defined by failure of spontaneous birth of the fetal shoulder after birth of the head, requiring obstetric manoeuvres [6]. Shoulder dystocia can result in neonatal morbidity (e.g. brachial plexus injury, fracture of humerus and clavicle, cerebral hypoxia) and mortality [6], and maternal morbidity (e.g. postpartum haemorrhage, perineal trauma) [6]. Its detrimental implications on the mother also include psychological consequences requiring counselling and emotional support [7] and increased obstetric intervention in subsequent pregnancies. For mothers and maternity care providers, shoulder dystocia is viewed as an ‘obstetric nightmare’ [8], and midwives in Australia and New Zealand rank it as the most feared of the obstetric emergencies [9]. Obstetric guidelines typically aim to reduce the risk of shoulder dystocia during childbirth by recommending caesarean section for women with DIP when the estimated fetal weight exceeds certain thresholds and by offering birth initiation for women with GDM after reaching a certain gestational age [10].

We previously reported that pre-gestational diabetes and GDM heightened the risk of shoulder dystocia by about 4.5- and three-fold, respectively, in Aboriginal babies [11]. In this paper, we hypothesized that investigating shoulder dystocia in Aboriginal women with DIP can identify specific gaps and help inform practice and guide preventive measures. The aim of this study was to investigate the epidemiology of shoulder dystocia in Aboriginal (relative to non-Aboriginal) babies born to mothers with DIP. We described the time trends of shoulder dystocia; explored the level of compliance with clinical guidelines aimed at preventing its occurrence; estimated the population attributable fraction (PAF) associated with DIP for shoulder dystocia; and estimated the adjusted probabilities of shoulder dystocia at different birthweights.

## Methods

### Design, data sources and study population

This retrospective cohort study used Western Australian (WA) population health datasets including Midwives’ Notification System (MNS), Hospital Morbidity Data Collection (HMDC) and WA Registry of Births, Deaths and Marriages. Datasets were linked by the Western Australian Department of Health data-linkage team, using probabilistic techniques [12]. The MNS, which is the main source of data used in this study, contains neonatal and maternal information recorded by midwives on the circumstances on all births that occur in WA. The study population included all singleton births that occurred in WA (*n* = 510,761) between 1998–2015 (Figure S1).

### Variables

#### Aboriginal status

Aboriginal status was identified using the Indigenous status flag created by the WA Department of Health to generate a single Aboriginal status for each individual in the linked administrative datasets. This approach used the algorithm of the ‘Getting Our Story Right’ project [13] with the aim of supporting more complete and consistent identification of the Aboriginal people in Western Australian state-wide datasets. The term 'Aboriginal' was used because the vast majority (96%) of the Aboriginal and Torres Strait Islander population in the state of Western Australia identify as solely Aboriginal.

#### Outcome and exposures

Shoulder dystocia was ascertained from the MNS. The condition is reported when there is delay and difficulty in delivering the fetal anterior shoulder that needed procedural interventions [14]. DIP was defined as the presence of pre-existing diabetes or GDM in either the MNS (under medical conditions or pregnancy complications) or HMDC data. In the HMDC data, DIP was captured using the relevant diagnostic codes (pre-existing diabetes: ICD-9-AM code 250, ICD-10-AM codes E10-11, E13-14, O24.0–42.3; GDM: ICD-9-AM code 648.8, ICD-10-AM codes O24.4, O24.9).

#### Covariates

Large-for-gestational-age (LGA) was defined as birthweight above the ninetieth percentile and appropriate-for-gestational-age (AGA) as birthweight between the tenth and ninetieth percentiles, using Australian gestational age- and sex-specific birthweight percentiles [15]. The relative geographic isolation of mothers (relative remoteness of residence) was determined using the Accessibility/Remoteness Index of Australia which classifies relative isolation into five categories, ranging from metropolitan to very remote [16]. We categorized areas of residence as remote or very remote and non-remote (if metropolitan, inner regional or outer regional). Hospital or place of birth was classified as metropolitan (included tertiary metropolitan public and private hospitals), rural (included public and private rural hospitals) and other birth site. The Index of Relative Socioeconomic Disadvantage [17] was used to determine socioeconomic status (categorized into tertiles). Parity was categorized into 0, 1, 2, or 3 or above. All births to the same mother in the MNS were linked to identify history of previous shoulder dystocia in multiparous women.

The levels of missing data were below 0.5% for all variables, except for maternal height (7.7%), socioeconomic status (4.1%) and remoteness (1.6%).

### Statistical analysis

Maternal and fetal characteristics of pregnancies delivered vaginally and complicated by shoulder dystocia were compared between those with and without DIP in analyses stratified by Aboriginal status. Pearson’s Chi-squared and Wilcoxon rank-sum tests compared categorical and continuous variables, respectively.

In analyses restricted to vaginal births, we described the time trends of shoulder dystocia by dividing the study period into four intervals (1998–2001, 2002–2005, 2006–2010 and 2011–2015), and comparing the incidence of shoulder dystocia over these periods in the Aboriginal and non-Aboriginal pregnancies with and without DIP.

The contribution of DIP to the burden of shoulder dystocia in the Aboriginal and non-Aboriginal populations was estimated by calculating the population attributable fractions (PAFs) in analyses restricted to vaginal deliveries. PAFs were estimated from multivariable logistic regression models adjusted for possible confounders (maternal age (continuous), parity [18] and remoteness [19] (binary)).PAFs were calculated using Greenland and Drescher methods [20] by comparing an assumed fantasy scenario where the exposure is set to zero (no DIP) with the observed distribution of DIP in the population.

We explored whether there were shoulder dystocia-related differences between the Aboriginal and non-Aboriginal populations in compliance with the local obstetric guidelines. The guidelines recommend considering elective caesarean section in women with DIP if the estimated fetal weight is above 4.5 kg [10]. The guidelines also state that delivery may be offered after 38 weeks in GDM pregnancies with a normally growing fetus [10]. We used Pearson’s Chi-squared test to compare (in the Aboriginal vs. non-Aboriginal mothers): the elective caesarean section rates in pregnancies with diabetes when birthweight is above 4.5 kg; and the proportions of initiated births (includes both induced and pre-labour caesarean births) at > 38 weeks in pregnancies with GDM and AGA babies.

The adjusted probabilities (and their 95% confidence intervals) of shoulder dystocia in pregnancies delivered vaginally and complicated by diabetes at birthweights > 3 kg were estimated after fitting multivariable logistic regression models. The models included birthweight (in continuous form), maternal age, parity group and remoteness (in the forms used in PAF models above) as independent variables and shoulder dystocia as the outcome variable. Locally weighted scatterplot smoothing suggested a linear relationship between birthweight (> 3 kg) and the log-odds of the occurrence of shoulder dystocia. Thus, we did not add polynomial terms to the multivariable regression models.

To account for the clustering effect of mothers who had multiple birth events in the longitudinal study cohort (which might result in biased estimates), robust standard error estimation was used in all multivariable models. Stata version 15.1 (StataCorp 2017) was used for statistical analysis.

### Aboriginal involvement

The design of this study and the interpretation of its findings were guided by the Kaadaninny Aboriginal Advisory Committee and Ngangk Yira Council of Elders. Ngangk Yira Institute for Change provided broader Cultural Governance. These groups will remain involved in future dissemination, communications and translational work.

## Results

Over the study period, Aboriginal births represented 6.4% (*n* = 32,845) of total births. There were 2,773 (8.5%) and 31,269 (6.6%) DIP cases among Aboriginal and non-Aboriginal births respectively (${x}^{2}$ = 177.05, *p < *0.001). In Aboriginal mothers with DIP, 6.3% of vaginal births were complicated by shoulder dystocia (99 cases), as compared to 3.2% (569 cases) in non-Aboriginal women with DIP (${x}^{2}$ = 40.85, *p < *0.001).

As shown in Table S1, Aboriginal women in the study cohort were more likely than non-Aboriginal women to be younger, smoke during pregnancy, reside in remote areas, have higher parity and be in the lower socioeconomic tertile while their infants were more likely to be SGA. Compared to those without DIP and regardless of Aboriginal status (Table S1), women with DIP were older; more likely to give births at earlier gestational ages; more likely to have LGA babies, induction of labour, caesarean section births; and less likely to smoke during pregnancy. Aboriginal and non-Aboriginal pregnancies with diabetes had comparable rates of labour induction and caesarean deliveries (39.9% vs 40.5%, ${x}^{2}$ = 0.40, *p* = 0.530). Aboriginal women with DIP had higher rates of emergency caesareans (59.1%) and lower rates of elective caesareans (40.9%) than non-Aboriginal women with DIP (45.9% and 54.1% for emergency and elective caesareans respectively, ${x}^{2}$ = 76.01, *p < *0.001).

When comparing pregnancies with diabetes complicated by shoulder dystocia, Aboriginal pregnancies were characterized by higher rates of previous shoulder dystocia; higher birthweights; younger gestational age; higher rates of LGA and lower rates of instrumental deliveries, relative to their non-Aboriginal counterparts (Table 1).

**Table 1:** Maternal and fetal characteristics of singleton pregnancies with and without diabetes complicated by shoulder dystocia, by Aboriginal status (restricted to vaginal births)

Characteristics	Aboriginal				Non-Aboriginal				Diabetes: Aboriginal vs non-Aboriginal	
	**No diabetes (*n* = 426)**	**Diabetes (*n* = 99)**	**Pearson’s Chi Square value**	***p*-value**^a^	**No diabetes (*n* = 6655)**	**Diabetes (*n* = 569)**	**Pearson’s Chi Square value**	***p*-value**^a^	**Pearson’s Chi Square value**	***p*-value**^a^
**Maternal age, median (IQR)**	24 (20, 29)	28 (24, 33)	-	< 0.001	29 (25, 33)	30 (27, 34)	-	< 0.001	-	0.001
**Maternal age group**										
** 25 or below**	254 (59.6%)	34 (34.3%)	27.96	< 0.001	1793 (26.9%)	91 (16.0%)	44.98	< 0.001	18.68	< 0.001
** > 25 to 35**	153 (35.9%)	50 (50.5%)			4064 (61.1%)	371 (65.2%)				
** above 35**	19 (4.5%)	15 (15.2%)			798 (12.0%)	107 (18.8%)				
**Maternal height (cm), median (IQR)**	163 (160, 167)	163 (158, 167)	-	0.360	164 (160, 168)	163 (157, 167)	-	< 0.001	-	0.810
**Birthweight (g), median (IQR)**	3900 (3610, 4280)	3990 (3590, 4390)	-	0.210	3960 (3680, 4270)	3845 (3540, 4150)	-	< 0.001	-	0.006
**Birthweight category**										
** below 3500**	82 (19.2%)	19 (19.2%)	6.49	0.370	986 (14.8%)	128 (22.5%)	42.93	< 0.001	20.53	0.002
** 3500 to 3750**	75 (17.6%)	17 (17.2%)			1068 (16.1%)	121 (21.3%)				
** 3750 to 4000**	83 (19.5%)	14 (14.1%)			1538 (23.1%)	113 (19.9%)				
** 4000 to 4250**	74 (17.4%)	12 (12.1%)			1332 (20.0%)	98 (17.2%)				
** 4250 to 4500**	59 (13.8%)	20 (20.2%)			944 (14.2%)	60 (10.5%)				
** 4500 to 4750**	26 (6.1%)	7 (7.1%)			484 (7.3%)	31 (5.4%)				
** 4750 and above**	27 (6.3%)	10 (10.1%)			303 (4.6%)	18 (3.2%)				
**Gestational age, median (IQR)**	40 (39, 40)	38 (37, 39)	-	< 0.001	40 (39, 40)	39 (38, 40)	-	< 0.001	-	0.014
**Gestational age category**										
** 34–35**	0 (0.0%)	< 6 (< 6.1%)^b^		< 0.001^c^	14 (0.2%)	12 (2.1%)	376.51	< 0.001	8.60	0.035
** 36–37**	20 (4.7%)	< 26 (< 26.3%)^b^			210 (3.2%)	84 (14.8%)				
** 38–39**	169 (39.9%)	47 (47.5%)			2540 (38.2%)	326 (57.5%)				
** 40 and above**	235 (55.4%)	23 (23.2%)			3882 (58.4%)	145 (25.6%)				
**Parity group**										
** 0**	122 (28.6%)	15 (15.2%)	16.15	0.001	2574 (38.7%)	202 (35.5%)	20.97	< 0.001	63.90	< 0.001
** 1**	109 (25.6%)	17 (17.2%)			2478 (37.2%)	185 (32.5%)				
** 2**	78 (18.3%)	24 (24.2%)			1055 (15.9%)	109 (19.2%)				
** 3 and above**	117 (27.5%)	43 (43.4%)			548 (8.2%)	73 (12.8%)				
**Smoking during pregnancy**	151 (35.4%)	33 (33.3%)	0.16	0.690	773 (11.6%)	77 (13.5%)	1.86	0.170	24.04	< 0.001
**Socioeconomic status**										
** 1 (most disadvantaged)**	300 (70.1%)	77 (83.7%)	5.25	0.072	2339 (36.6%)	238 (43.5%)	10.36	0.006	53.68	< 0.001
** 2**	60 (15.4%)	< 16 (< 16.2%)^b^			2252 (35.3%)	176 (32.2%)				
** 3**	29 (7.5%)	< 6 (< 6.1%)^b^			1793 (28.1%)	133 (24.3%)				
**Remote residence**	195 (46.9%)	51 (52.0%)	0.85	0.360	665 (10.2%)	45 (8.0%)	2.66	0.100	130.17	0.001
**Type of hospital**										
** Metro hospital**	148 (34.7%)	39 (39.4%)	0.76	0.380	4887 (73.8%)	471 (82.8%)	25.20	< 0.001	87.89	< 0.001
** Rural hospital**	278 (65.3%)	60 (60.6%)			1734 (26.2%)	98 (17.2%)				
** Other birth site**	0 (0.0%)	0 (0.0%)			34 (0.5%)	0 (0.0%)				
**Sex (female)**	183 (43.0%)	41 (41.4%)	0.08	0.780	2854 (42.9%)	269 (47.3%)	4.12	0.042	1.17	0.280
**Large for gestational age**	162 (38.1%)	62 (62.6%)	19.70	< 0.001	2629 (39.5%)	245 (43.1%)	2.74	0.098	13.00	< 0.001
**Appropriate for gestational age**	261 (61.4%)	36 (36.4%)	20.52	< 0.001	3981 (59.8%)	320 (56.2%)	2.83	0.092	13.38	< 0.001
**Previous shoulder dystocia**	23 (5.4%)	11 (11.1%)	4.33	0.038	244 (3.7%)	23 (4.0%)	0.21	0.650	8.72	0.003
**Previous shoulder dystocia (restricted to parous women)**	23 (7.6%)	11 (13.1%)	2.51	0.110	244 (6.0%)	23 (6.3%)	0.05	0.820	4.57	0.032
**Method of birth**										
** Spontaneous vaginal**	342 (80.3%)	86 (86.9%)	2.31	0.130	4459 (67.0%)	375 (65.9%)	0.29	0.590	17.33	< 0.001
** Instrumental vaginal**	84 (19.7%)	13 (13.1%)			2196 (33.0%)	194 (34.1%)				
**No labour induction**	294 (69.0%)	43 (43.4%)	22.87	< 0.001	4097 (61.6%)	201 (35.3%)	149.74	< 0.001	2.39	0.210
**Type of labour induction**										
** Induction with oxytocin**	103 (78.0%)	45 (80.4%)	0.13	0.721	1966 (77.9%)	276 (75.0%)	0.62	0.431	0.76	0.384
** Other induction method**	29 (22.0)	11 (19.6%)			592 (23.1%)	92 (25.0%)				

*IQR* Interquartile range

^a^Pearson's chi-squared test (or Fisher's exact test) p-values for categorical variables and Wilcoxon–Mann–Whitney test p-values for continuous variables

^b^The numbers (percentages) for small cells were not shown to maintain confidentiality. To prevent the calculation of numbers in small groups, the numbers (percentages) of the second smallest groups are not presented

^c^Fisher's exact test *p*-value

**Fig. 1:**
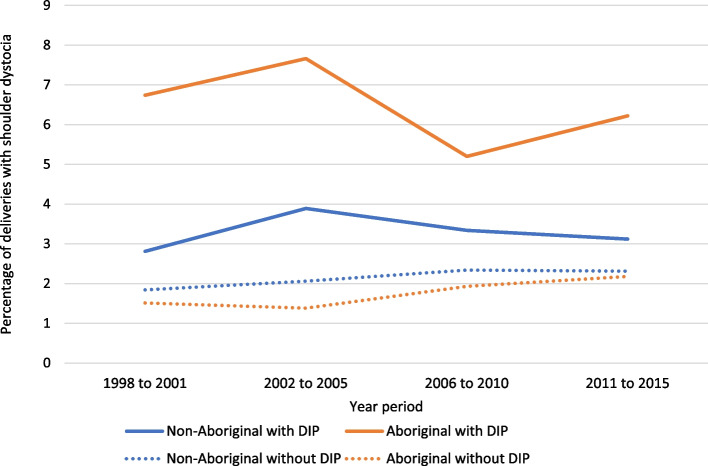
Rates of shoulder dystocia over time in singleton pregnancies with and without diabetes, by Aboriginal status (restricted to vaginal births). DIP: diabetes in pregnancy

**Fig. 2:**
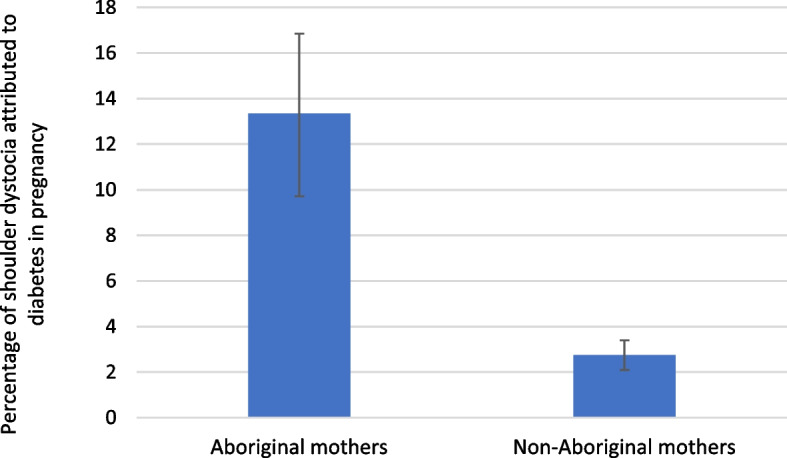
Adjusted^a^ population attributable fraction for shoulder dystocia associated with diabetes in pregnancy among the Aboriginal and non-Aboriginal populations (restricted to vaginal singleton births). ^a^Adjusted for maternal age (continuous), parity (categorical) and remoteness (binary)

The disparities between the Aboriginal and non-Aboriginal populations in the incidence of shoulder dystocia in pregnancies complicated by diabetes did not decrease over the study period (Fig. 1). The PAFs indicate that 13.4% (95% CI: 9.7–16.9) of shoulder dystocia cases in the Aboriginal population were attributed to DIP compared to 2.7% (95% CI: 2.1–3.4) in the non-Aboriginal population (Fig. 2).

As for compliance with the guidelines, Table S2 shows that Aboriginal pregnancies with DIP and birthweight > 4.5 kg had lower rates of elective caesarean section (28.6%) when compared to their non-Aboriginal counterparts (43.1%) (${x}^{2}$= 8.29, *p* = 0.004). The rates of initiated births among GDM pregnancies with AGA babies at > 38 weeks were relatively lower in Aboriginal mothers (Aboriginal: 58.6% vs. non-Aboriginal: 62.8%, ${x}^{2}$ = 4.03, *p* = 0.045) (Table S3).

**Fig. 3:**
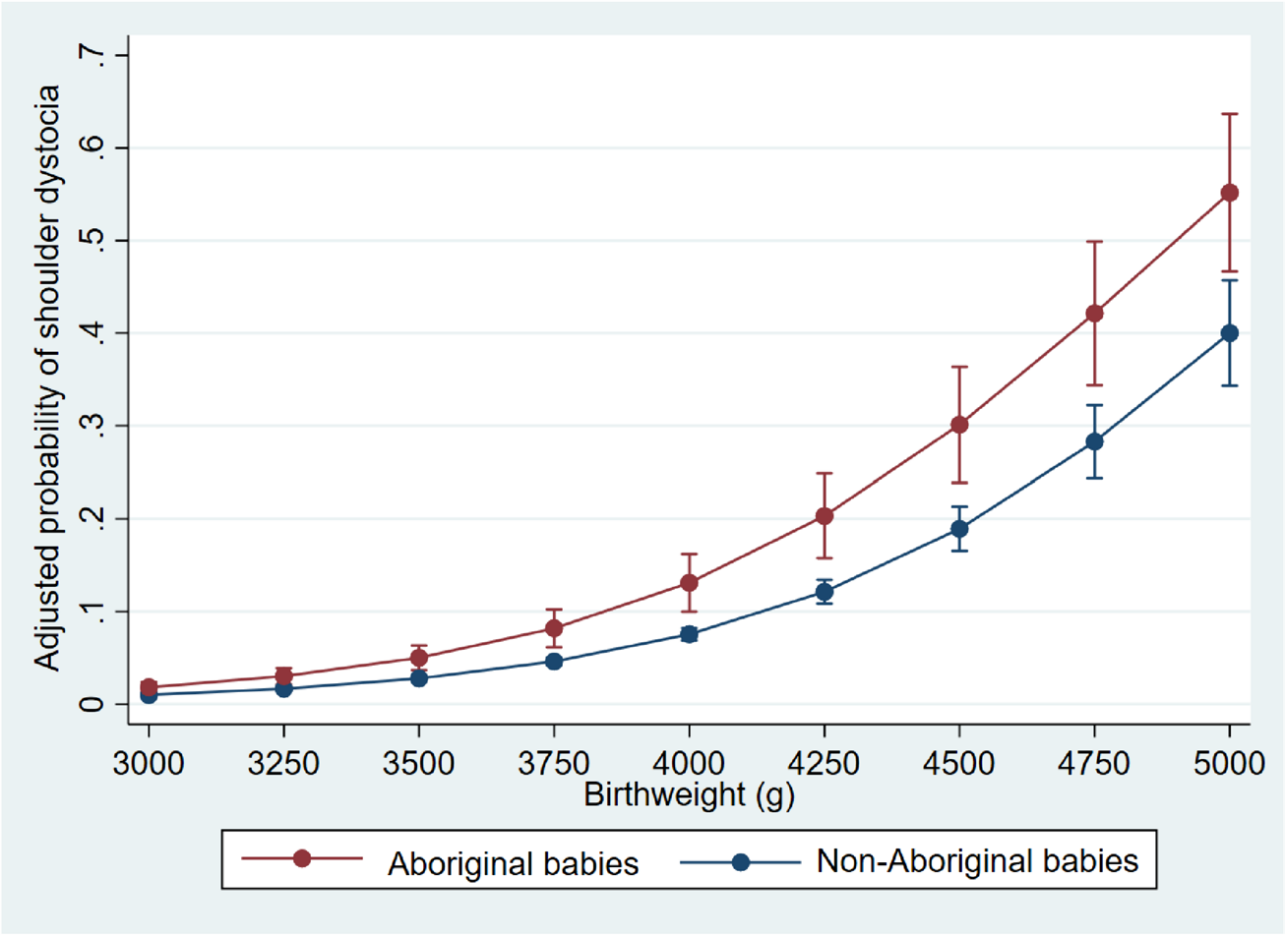
Adjusted^a^ predicted probability of shoulder dystocia in diabetic pregnancies delivered vaginally by birthweight and Aboriginal status. ^a^Adjusted for maternal age (continuous), parity (categorical) and remoteness (binary)

Among pregnancies complicated by diabetes, the adjusted probability of shoulder dystocia was higher in Aboriginal babies born vaginally at birthweights above 3 kg, and the difference between the two populations appears to increase with birthweight (Fig. 3).

## Discussion

### Summary of the findings

This study reported greater rates of shoulder dystocia in Aboriginal babies born to mothers with diabetes (6.3% vs. 3.2% in non-Aboriginal babies), and the disparities have not improved with time. About 13.4% of shoulder dystocia in the Aboriginal population (2.7% in non-Aboriginal population) was attributable to DIP. The adjusted probabilities of shoulder dystocia in Aboriginal babies than non-Aboriginal babies were higher at birthweights above 3 kg, with the gap between the two populations increasing with birthweight. Multiparous Aboriginal mothers with diabetes whose births were complicated by shoulder dystocia were more likely to have a previous history of shoulder dystocia.

There appeared to be lower compliance with the guidelines aiming at reducing the occurrence of shoulder dystocia among Aboriginal pregnancies with diabetes than in non-Aboriginal mothers. Aboriginal mothers with DIP were less likely to have an elective caesarean section when their babies had birthweights > 4.5 kg compared to their non-Aboriginal counterparts. Aboriginal mothers with GDM also had lower rates of initiated births after 38 completed weeks of gestation.

### Shoulder dystocia in mothers with diabetes and birthweight

In Aboriginal babies born to mothers with diabetes, the stronger association between birthweight and shoulder dystocia and the higher proportions of shoulder dystocia cases at high birthweight categories point to a key role for excessive fetal growth in the occurrence of this outcome. DIP is a well-established risk factor for shoulder dystocia through pathways that involve increasing fetal weight and changing fetal anthropometry. Maternal hyperglycemia increases fetal weight linearly without a threshold [21] by stimulating fetal hyperinsulinemia that stimulates protein synthesis and lipid deposition [22]. Moreover, maternal hyperglycemia results in disproportionate fetal growth. Infants born to mothers with diabetes have thicker skinfold of upper extremities and higher shoulder-to-head and chest-to-head ratios [23–25]. Therefore, maternal diabetes increases the risk of shoulder dystocia independent of macrosomia and at birthweights below 4 kg [25, 26]. We thus believe that excessive fetal growth and fetal disproportion (both mediated by suboptimal glycemia) are the drivers of the heavier burden of shoulder dystocia in the Aboriginal babies born to mothers with diabetes, and of the stronger association between birthweight and shoulder dystocia in these babies. Previous studies have indicated that Aboriginal Australians with diabetes are more likely to have glycaemic levels above the target range [27, 28], and this is consistent with poorer access and late presentation for antenatal care [29].

Our study has implications on practice and future research. The considerably higher rates of shoulder dystocia in babies born to Aboriginal mothers with diabetes and their persistence over time point to the need for change. Although current practice recognises DIP as a risk factor for shoulder dystocia [10], the findings emerging from this study suggest considering Aboriginal women with diabetes as a subpopulation at a further heightened risk of shoulder dystocia. Based on the finding of the higher probability of shoulder dystocia along the birthweight continuum in babies born to Aboriginal mothers with diabetes, clinicians may offer these mothers caesarean sections at estimated fetal weights lower than 4.5 kg (which is the threshold in current practice regardless of the treatment status of diabetes [10]). Importantly, there are also recommendations on taking 4.25 kg as the threshold for caesarean section in mothers with DIP [30, 31]. Moreover, Aboriginal women with DIP should be counselled regarding risk of shoulder dystocia and available preventive strategies. Our study reveals the important role whole-population, though administrative, datasets can play in uncovering and investigating relatively infrequent, but important and preventable, obstetric complications. We believe this study could be replicated in different, culturally distinct, Aboriginal populations in Australia. High DIP rates [3], high glycemic levels [28], limited access to antenatal care [29] and lower rates of caesarean section [32] have all been reported among Aboriginal Australian populations. Whole-population data, now available in all Australian states/territories, can provide a sufficiently powered tool to investigate this obstetric complication.

Although the effect of hyperglycemia was not directly investigated in this study, our findings suggest a substantial impact of suboptimal glycemic levels in Aboriginal women with DIP and highlight the need for improving access to appropriate antenatal care for these women. Real-world research, quantitative and qualitative, undertaken in partnership with Aboriginal community members is needed to identify the challenges towards achieving euglycemia in these high-risk pregnancies.

### History of shoulder dystocia and compliance with guidelines

Previous shoulder dystocia is an established risk factor for shoulder dystocia [33]. The higher recurrence of shoulder dystocia in Aboriginal mothers, who are generally known to have considerably higher parity, may indicate missed opportunities for prevention. In non-Aboriginal women, pregnancies subsequent to those complicated by shoulder dystocia probably tended to have caesarean deliveries, avoiding recurrence. Due attention should thus be paid to previous events of shoulder dystocia when caring for Aboriginal pregnancies complicated by diabetes.

The lower rates of elective caesarean in Aboriginal women with DIP and infant birthweight above 4.5 kg may be a consequence of suboptimal access to antenatal care or an artefact of differences in culture and preference with the non-Aboriginal mothers. These low rates indicate the need for improving the compliance with guidelines, probably through clinician education and patient counselling about the risk of shoulder dystocia.

Clinical decisions around labour induction in pregnancies complicated by GDM are complex and rely largely on the treatment status of diabetes and maternal glycemic control. Since datasets used in this paper do not include information on these factors, we cannot draw conclusions about the findings of the lower proportions of initiated births in the Aboriginal, compared to non-Aboriginal, mothers with GDM at gestational ages above 38 weeks.

### Strengths and limitations

Using population-wide data collected from different sources is a strength of this study. This provides sufficient power to detect infrequent outcomes, removes selection bias, maximizes generalisability and prevents loss to follow up upon transfer between healthcare facilities.

Our study has limitations, mainly related to data availability. Absence of important variables precluded proper explanations and probably impacted on the accuracy of the reported estimates. Our proposed hyperglycemia- and fetal anthropometry-mediated stronger association between birthweight and probability of shoulder dystocia in the Aboriginal, relative to non-Aboriginal, mothers with DIP was not based on relevant data (due to the absence of information on glycemic biomarkers, treatment of diabetes and fetal anthropometric measurements). Moreover, the datasets lack information on the timing and results of ultrasound scans. We thus used actual birthweight as a proxy for the estimated fetal weight (on which decisions around delivery are based). The ultrasound-based estimated fetal weight, which may have an error margin of up to 15% [34], can result in misclassification of fetal weight category (above or below 4.5 kg), and the status of compliance with the guidelines can thus be misclassified. Despite some preliminary research [35, 36], we could not find any current reliable tools to accurately predict fetal macrosomia. Our data also lack information on maternal overweight/obesity, an independent risk factor for shoulder dystocia [37], that may explain, at least, part of its elevated risk in Aboriginal mothers with DIP. Recent evidence has shown suboptimal rates of screening for GDM in remote and rural Western Australia [38]. Aboriginal women, who are more likely to live in remote areas, may thus have a different severity profile of GDM (screening may have differentially captured Aboriginal mothers with more severe hyperglycemia), biasing the reported estimates. It is likely that this impact was minimized by adjusting for remoteness in the multivariable analyses. The new guidelines for the diagnosis of GDM published by the Australasian Diabetes in Pregnancy Society [39] have resulted in increased GDM prevalence [5], and may have captured milder cases of GDM and thus resulted in lower rates of shoulder dystocia. However, to our knowledge, there is no evidence on a differential impact of the new guidelines on the prevalence and perinatal outcomes of GDM in the Aboriginal, relative to non-Aboriginal, populations.

## Conclusions

DIP heightened the risk of shoulder dystocia to a greater extent in Aboriginal compared to non-Aboriginal babies. This differential heightened risk is possibly explained by differences in fetal size (driven by suboptimal glycemic control) and the delivery of clinical care for pregnancies complicated by diabetes. Babies born to Aboriginal mothers with diabetes had higher probabilities of shoulder dystocia compared to their non-Aboriginal counterparts who had similar birthweights, and the difference between the two groups grew as birthweight increased. Clinical practice guidelines should reflect this higher risk, and due attention should be given to previous events of shoulder dystocia in Aboriginal mothers. Our findings highlight the importance of optimal diabetes management for pregnant Aboriginal women and the need to identify strategies to strengthen appropriate, effective care for DIP. We also recommend studies to investigate shoulder dystocia among Aboriginal mothers with diabetes in other Australian states/territories.

### Supplementary Information

**Supplementary Material 1.**

**Supplementary Material 2.**

**Supplementary Material 3.**

**Supplementary Material 4.**

## Data Availability

The data used in this paper are not publicly available and cannot be provided by the authors due to restrictions by the data custodians. To have access to the data, researchers should refer to the Data Linkage System of the Western Australia Government Department of Health (www.datalinkage-wa.org.au). Upon reasonable request and with the express permission of the Western Australian DoH Human Research Ethics Committee and Western Australian Aboriginal Health Ethics Committee, the corresponding author is willing to make every effort to grant data availability.

## Abbreviations

AGA: Appropriate-for-gestational-age
DIP: Diabetes in pregnancy
GDM: Gestational diabetes mellitus
HMDC: Hospital Morbidity Database Collection
LGA: Large for gestational age
MNS: Midwives Notification System
PAF: Population attributable fraction
