# Supplementary figures and images for: Shoulder dystocia in babies born to Aboriginal mothers with diabetes: a population-based cohort study, 1998–2015
Source: BMC Pregnancy Childbirth. 2024 May 30;24:395. doi: 10.1186/s12884-024-06484-1 (PMC11137982; doi:10.1186/s12884-024-06484-1)

Figure S1: Flowchart of the study population


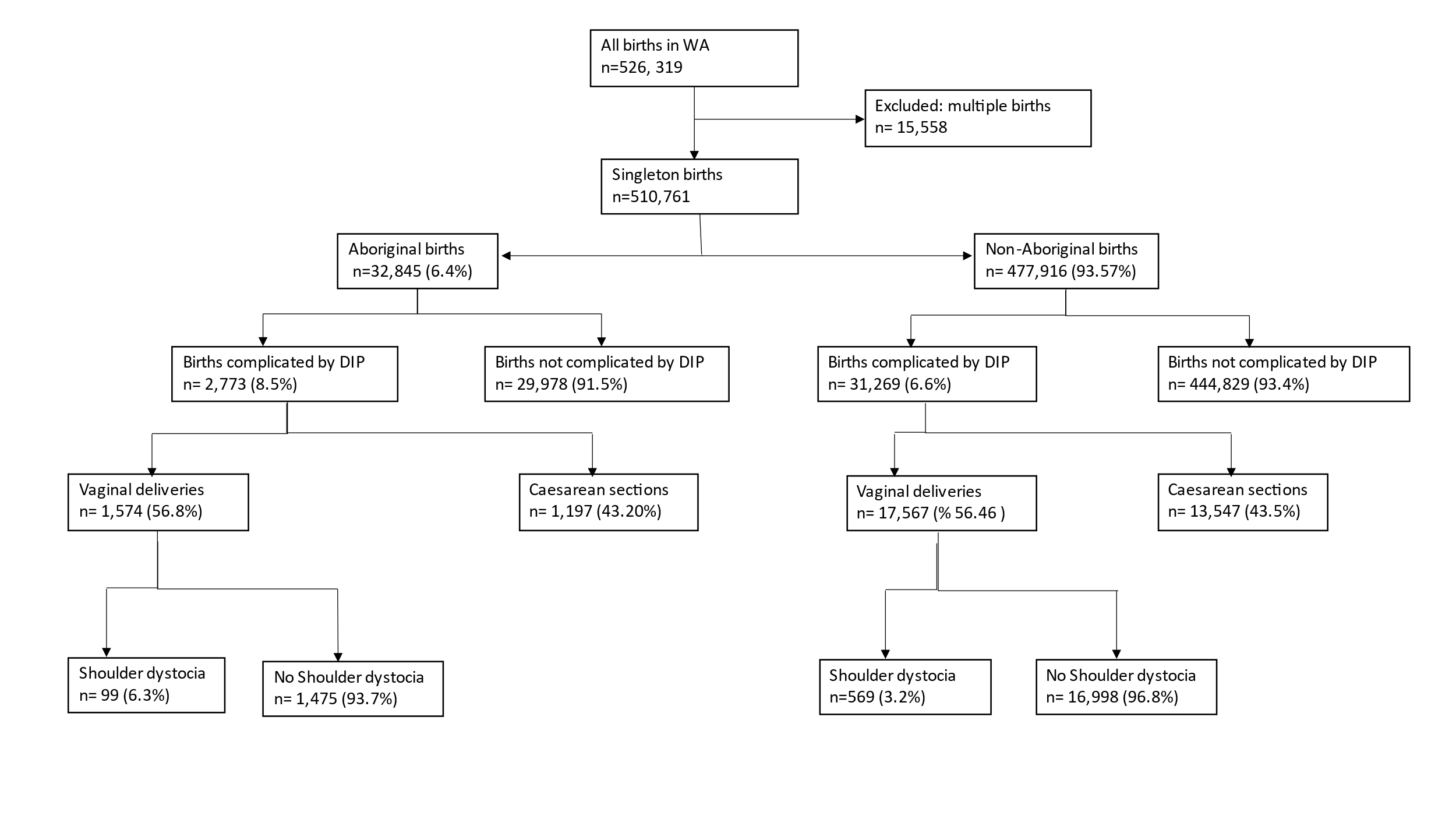

Supplement: Supplementary file 1 — Supplementary Material 1. [file 12884_2024_6484_MOESM1_ESM.docx]
