# Supplementary material for: Shoulder dystocia in babies born to Aboriginal mothers with diabetes: a population-based cohort study, 1998–2015
Source: BMC Pregnancy Childbirth. 2024 May 30;24:395. doi: 10.1186/s12884-024-06484-1 (PMC11137982; doi:10.1186/s12884-024-06484-1)
Supplement: Supplementary file 2 — Supplementary Material 2. [file 12884_2024_6484_MOESM2_ESM.docx]

Table S1: Maternal and fetal characteristics of singleton pregnancies with and without diabetes, by Aboriginal status

| **Characteristics** | | **Aboriginal** | | | | **Non-Aboriginal** | | | | **Diabetes: Aboriginal vs non-Aboriginal** | |
| --- | --- | --- | --- | --- | --- | --- | --- | --- | --- | --- | --- |
|  |  | **No diabetes**  **(n=29978)** | **Diabetes**  **(n=2773)** | **Pearson's Chi Square value** | **p-value^a^** | **No diabetes**  **(n=444829)** | **Diabetes**  **(n=31269)** | **Pearson's Chi Square value** | **p-value^a^** | **Pearson's Chi Square value** | **p-value^a^** |
| **Diabetes in pregnancy** | |  | 2773 (8.5%^b^) |  |  |  | 31269 (6.6%^c^) |  |  | 177.05 | <0.001 |
| **Maternal age, median (IQR)** | | 23 (20, 28) | 29 (24, 34) | - | <0.001 | 30 (26, 34) | 32 (28, 36) | - | <0.001 | - | <0.001 |
| **Maternal age group** | |  |  |  |  |  |  |  |  |  |  |
|  | **25 or below** | 18779 (62.6%) | 900 (32.5%) | 1321.43 | <0.001 | 102709 (23.1%) | 3596 (11.5%) | 3996.23 | <0.001 | 988.26 | <0.001 |
|  | **>25 to 35** | 9890 (33.0%) | 1414 (51.0%) |  |  | 277032 (62.3%) | 19709 (63.0%) |  |  |  |  |
|  | **above 35** | 1309 (4.4%) | 459 (16.6%) |  |  | 65088 (14.6%) | 7964 (25.5%) |  |  |  |  |
| **Maternal height (cm), median (IQR)** | | 163 (159, 167) | 163 (159, 166) | - | 0.013 | 165 (160, 170) | 163 (158, 167) | - | <0.001 | - | 0.950 |
| **Birthweight (g), median (IQR)** | | 3180 (2790, 3550) | 3410 (2960, 3800) | - | <0.001 | 3410 (3090, 3730) | 3380 (3040, 3720) | - | <0.001 | - | 0.440 |
| **Birthweight category** | |  |  |  |  |  |  |  |  |  |  |
|  | **below 3500** | 21604 (72.1%) | 1566 (56.5%) | 546.50 | <0.001 | 257609 (57.9%) | 18720 (59.9%) | 152.69 | <0.001 | 113.62 | <0.001 |
|  | **3500 to 3750** | 3884 (13.0%) | 438 (15.8%) |  |  | 82826 (18.6%) | 5387 (17.2%) |  |  |  |  |
|  | **3750 to 4000** | 2451 (8.2%) | 309 (11.1%) |  |  | 56660 (12.7%) | 3644 (11.7%) |  |  |  |  |
|  | **4000 to 4250** | 1224 (4.1%) | 200 (7.2%) |  |  | 29212 (6.6%) | 1969 (6.3%) |  |  |  |  |
|  | **4250 to 4500** | 531 (1.8%) | 147 (5.3%) |  |  | 12317 (2.8%) | 941 (3.0%) |  |  |  |  |
|  | **4500 to 4750** | 177 (0.6%) | 66 (2.4%) |  |  | 4378 (1.0%) | 395 (1.3%) |  |  |  |  |
|  | **4750 and above** | 106 (0.4%) | 47 (1.7%) |  |  | 1821 (0.4%) | 207 (0.7%) |  |  |  |  |
|  | |  |  |  |  |  |  |  |  |  |  |
| **Gestational age, median (IQR)** | | 39 (38, 40) | 38 (37, 39) | - | <0.001 | 39 (38, 40) | 38 (37, 39) | - | <0.001 | - | <0.001 |
| **Gestational age category** | |  |  |  |  |  |  |  |  |  |  |
|  | **34-35** | 1217 (4.3%) | 199 (7.6%) | 473.58 | <0.001 | 9195 (2.1%) | 1181 (3.9%) | 6101.37 | <0.001 | 174.39 | <0.001 |
|  | **36-37** | 4416 (15.5%) | 684 (26.3%) |  |  | 46186 (10.6%) | 5909 (19.4%) |  |  |  |  |
|  | **38-39** | 13213 (46.3%) | 1304 (50.1%) |  |  | 216848 (49.7%) | 18004 (59.2%) |  |  |  |  |
|  | **40 and above** | 9676 (33.9%) | 416 (16.0%) |  |  | 164254 (37.6%) | 5307 (17.5%) |  |  |  |  |
| **Parity group** | |  |  |  |  |  |  |  |  |  |  |
|  | **0** | 9188 (30.6%) | 535 (19.3%) | 315.06 | <0.001 | 188843 (42.5%) | 12956 (41.5%) | 187.84 | <0.001 | 2598.90 | <0.001 |
|  | **1** | 7237 (24.1%) | 558 (20.1%) |  |  | 154808 (34.8%) | 10393 (33.3%) |  |  |  |  |
|  | **2** | 5263 (17.6%) | 508 (18.3%) |  |  | 66556 (15.0%) | 4759 (15.3%) |  |  |  |  |
|  | **3 plus** | 8290 (27.7%) | 1171 (42.2%) |  |  | 34622 (7.8%) | 3082 (9.9%) |  |  |  |  |
| **Smoking during pregnancy** | | 14831 (49.5%) | 1205 (43.5%) | 36.38 | <0.001 | 60865 (13.7%) | 3194 (10.3%) | 291.55 | <0.001 | 2485.68 | <0.001 |
| **Socioeconomic status** | |  |  |  |  |  |  |  |  |  |  |
|  | **1 (most disadvantaged)** | 21319 (76.6%) | 1929 (76.2%) | 1.08 | 0.580 | 140379 (32.8%) | 10772 (35.6%) | 128.44 | <0.001 | 1687.97 | <0.001 |
|  | **2** | 4961 (17.8%) | 469 (18.5%) |  |  | 146501 (34.3%) | 10378 (34.3%) |  |  |  |  |
|  | **3** | 1536 (5.5%) | 132 (5.2%) |  |  | 140687 (32.9%) | 9139 (30.2%) |  |  |  |  |
| **Remote residence** | | 13038 (44.7%) | 1359 (50.5%) | 33.95 | <0.001 | 35712 (8.1%) | 1865 (6.0%) | 173.99 | <0.001 | 5635.64 | <0.001 |
| **Type of hospital** | |  |  |  |  |  |  |  |  |  |  |
|  | **Metro hospital** | 13814 (46.1%) | 1527 (55.1%) | 84.17 | <0.001 | 364293 (81.9%) | 27557 (88.1%) | 848.44 | <0.001 | 2263.06 | <0.001 |
|  | **Rural hospital** | 16136 (53.8%) | 1246 (44.9%) |  |  | 77251 (17.4%) | 3682 (11.8%) |  |  |  |  |
|  | **Other birth site** | 28 (0.1%) | 0 (0.0%) |  |  | 3285 (0.7%) | 30 (0.1%) |  |  |  |  |
| **Sex (female)** | | 14781 (49.3%) | 1320 (47.6%) | 2.96 | 0.086 | 217352 (48.9%) | 14988 (47.9%) | 10.11 | 0.001 | 0.11 | 0.740 |
| **Large for gestational age** | | 2096 (7.0%) | 736 (26.6%) | 1225.58 | <0.001 | 46443 (10.4%) | 5471 (17.6%) | 1526.07 | <0.001 | 137.43 | <0.001 |
| **Appropriate for gestational age** | | 22688 (75.9%) | 1806 (65.2%) | 153.83 | <0.001 | 362513 (81.5%) | 23397 (75.2%) | 755.36 | <0.001 | 133.15 | <0.001 |
| **Small for gestational age** | | 5111 (17.1%) | 227 (8.2%) | 146.78 | <0.001 | 35740 (8.0%) | 2241 (7.2%) | 27.48 | <0.001 | 3.72 | 0.054 |
| **Induction of labour** | | 6177 (20.6%) | 1105 (39.9%) | 544.84 | <0.001 | 127547 (28.7%) | 12598 (40.5%) | 1954.79 | <0.001 | 0.40 | 0.530 |
| **Caesarean delivery** | | 6142 (20.5%) | 1197 (43.2%) | 752.30 | <0.001 | 137691 (31.0%) | 13547 (43.5%) | 2124.92 | <0.001 | 0.12 | 0.730 |
| **Shoulder dystocia (restricting to vaginal deliveries)** | | 426 (1.8%) | 99 (6.3%) | 147.59 | <0.001 | 6655 (2.2%) | 569 (3.2%) | 83.98 | <0.001 | 40.85 | <0.001 |
| **Type of caesarean (restricting to caesarean deliveries)** | |  |  |  |  |  |  |  |  |  |  |
|  | **Emergency** | 3944 (64.2%) | 707 (59.1%) | 11.44 | <0.001 | 62321 (45.3%) | 6224 (45.9%) | 2.32 | 0.130 | 76.01 | <0.001 |
|  | **Elective** | 2198 (35.8%) | 490 (40.9%) |  |  | 75370 (54.7%) | 7323 (54.1%) |  |  |  |  |

IQR: Interquartile range

^a^Pearson's chi-squared test p-values for categorical variables and Wilcoxon–Mann–Whitney test p-values for continuous variables

^b^Percentage represents the number of Aboriginal pregnancies complicated by diabetes divided by the total number of Aboriginal pregnancies.

^c^Percentage represents the number of non-Aboriginal pregnancies complicated by diabetes divided by the total number of non-Aboriginal pregnancies.
