# Supplementary material for: Shoulder dystocia in babies born to Aboriginal mothers with diabetes: a population-based cohort study, 1998–2015
Source: BMC Pregnancy Childbirth. 2024 May 30;24:395. doi: 10.1186/s12884-024-06484-1 (PMC11137982; doi:10.1186/s12884-024-06484-1)
Supplement: Supplementary file 3 — Supplementary Material 3. [file 12884_2024_6484_MOESM3_ESM.docx]

Table S2: Rates of elective caesarean section in Aboriginal and non-Aboriginal mothers with diabetes in pregnancy and infant weight above 4.5 kg

|  |  | **Aboriginal mothers** | **Non-Aboriginal mothers** | **Pearson's Chi Square value** | **p-value** |
| --- | --- | --- | --- | --- | --- |
| **Elective caesarean section** | yes | 32 (28.6%) | 258 (43.1%) | 8.29 | 0.004 |
|  | no | 80 (71.4) | 340 (56.9) |  |  |
