# Supplementary material for: Shoulder dystocia in babies born to Aboriginal mothers with diabetes: a population-based cohort study, 1998–2015
Source: BMC Pregnancy Childbirth. 2024 May 30;24:395. doi: 10.1186/s12884-024-06484-1 (PMC11137982; doi:10.1186/s12884-024-06484-1)
Supplement: Supplementary file 4 — Supplementary Material 4. [file 12884_2024_6484_MOESM4_ESM.docx]

Table S3: Rates of initiated births in Aboriginal and non-Aboriginal singleton pregnancies complicated by gestational diabetes that completed 38 weeks restricting to appropriate for gestational age births

|  |  | **Aboriginal mothers** | **non-Aboriginal mothers** | **Pearson's Chi Square value** | **p-value** |
| --- | --- | --- | --- | --- | --- |
| **Initiated births** | yes | 327 (58.6%) | 6,151 (62.8%) | 4.03 | 0.045 |
|  | no | 231 (41.4%) | 3,639 (37.2%) |  |  |
